# Distance Management of Spinal Disorders During the COVID-19 Pandemic and Beyond: Evidence-Based Patient and Clinician Guides From the Global Spine Care Initiative

**DOI:** 10.2196/25484

**Published:** 2021-02-17

**Authors:** Scott Haldeman, Margareta Nordin, Patricia Tavares, Rajani Mullerpatan, Deborah Kopansky-Giles, Vincent Setlhare, Roger Chou, Eric Hurwitz, Caroline Treanor, Jan Hartvigsen, Michael Schneider, Ralph Gay, Jean Moss, Joan Haldeman, David Gryfe, Adam Wilkey, Richard Brown, Geoff Outerbridge, Stefan Eberspaecher, Linda Carroll, Reginald Engelbrecht, Kait Graham, Nathan Cashion, Stefanie Ince, Erin Moon

**Affiliations:** 1 World Spine Care Santa Ana, CA United States; 2 Faculty of Health Sciences University of Ontario Institute of Technology Oshawa, ON Canada; 3 Department of Neurology University of California Irvine, CA United States; 4 Department of Orthopedic Surgery New York University New York, NY United States; 5 Department of Environmental Medicine New York University New York, NY United States; 6 Department of Clinical Education Canadian Memorial Chiropractic College Toronto, ON Canada; 7 MGM School of Physiotherapy MGM Institute of Health Sciences Navi, Mumbai India; 8 Department of Research Canadian Memorial Chiropractic College Toronto, ON Canada; 9 Department of Family & Community Medicine University of Toronto Toronto, ON Canada; 10 Department of Family Medicine and Public Health Medicine University of Botswana Gaborone Botswana; 11 Department of Medicine Oregon Health & Science University Portland, OR United States; 12 Department of Medical Informatics & Clinical Epidemiology Oregon Health & Science University Portland, OR United States; 13 Office of Public Health Studies University of Hawaii Manoa, HI United States; 14 Department of Physiotherapy Beaumont Hospital Dublin Ireland; 15 Department of Neurosurgery National Neurosurgical Spinal Service Beaumont Hospital Dublin Ireland; 16 Department of Sports Science and Clinical Biomechanics University of Southern Denmark Odense Denmark; 17 Nordic Institute of Chiropractic and Clinical Biomechanics Odense Norway; 18 School of Health and Rehabilitation Sciences University of Pittsburgh Pittsburgh, PA United States; 19 Clinical and Translational Science Institute University of Pittsburgh Pittsburgh, PA United States; 20 Department of Physical Medicine and Rehabilitation Mayo Clinic Alix School of Medicine Rochester, MN United States; 21 Canadian Memorial Chiropractic College Toronto, ON Canada; 22 World Spine Care Europe Holmfirth United Kingdom; 23 World Federation of Chiropractic Toronto, ON Canada; 24 World Spine Care Ottawa, ON Canada; 25 School of Public Health University of Alberta Edmonton, AB Canada; 26 World Spine Care, South Africa Bethlehem South Africa; 27 Volunteer Programs and Operations World Spine Care Ottawa, ON Canada; 28 Digital Communications World Spine Care Oregon City, OR United States; 29 World Spine Care Toronto, ON Canada; 30 World Spine Care Yoga Project Vancouver, BC Canada

**Keywords:** COVID-19, spinal disorders, evidence-based care, physical distancing care, clinical guides, low- and middle-income communities, telehealth, telemedicine, evidence-based, spine, guide, management

## Abstract

**Background:**

The COVID-19 pandemic has greatly limited patients' access to care for spine-related symptoms and disorders. However, physical distancing between clinicians and patients with spine-related symptoms is not solely limited to restrictions imposed by pandemic-related lockdowns. In most low- and middle-income countries, as well as many underserved marginalized communities in high-income countries, there is little to no access to clinicians trained in evidence-based care for people experiencing spinal pain.

**Objective:**

The aim of this study is to describe the development and present the components of evidence-based patient and clinician guides for the management of spinal disorders where in-person care is not available.

**Methods:**

Ultimately, two sets of guides were developed (one for patients and one for clinicians) by extracting information from the published Global Spine Care Initiative (GSCI) papers. An international, interprofessional team of 29 participants from 10 countries on 4 continents participated. The team included practitioners in family medicine, neurology, physiatry, rheumatology, psychology, chiropractic, physical therapy, and yoga, as well as epidemiologists, research methodologists, and laypeople. The participants were invited to review, edit, and comment on the guides in an open iterative consensus process.

**Results:**

The Patient Guide is a simple 2-step process. The first step describes the nature of the symptoms or concerns. The second step provides information that a patient can use when considering self-care, determining whether to contact a clinician, or considering seeking emergency care. The Clinician Guide is a 5-step process: (1) Obtain and document patient demographics, location of primary clinical symptoms, and psychosocial information. (2) Review the symptoms noted in the patient guide. (3) Determine the GSCI classification of the patient’s spine-related complaints. (4) Ask additional questions to determine the GSCI subclassification of the symptom pattern. (5) Consider appropriate treatment interventions.

**Conclusions:**

The Patient and Clinician Guides are designed to be sufficiently clear to be useful to all patients and clinicians, irrespective of their location, education, professional qualifications, and experience. However, they are comprehensive enough to provide guidance on the management of all spine-related symptoms or disorders, including triage for serious and specific diseases. They are consistent with widely accepted evidence-based clinical practice guidelines. They also allow for adequate documentation and medical record keeping. These guides should be of value during periods of government-mandated physical or social distancing due to infectious diseases, such as during the COVID-19 pandemic. They should also be of value in underserved communities in high-, middle-, and low-income countries where there is a dearth of accessible trained spine care clinicians. These guides have the potential to reduce the overutilization of unnecessary and expensive interventions while empowering patients to self-manage uncomplicated spinal pain with the assistance of their clinician, either through direct in-person consultation or via telehealth communication.

## Introduction

This paper has been developed primarily in response to the impact of the COVID-19 pandemic caused by the SARS-CoV-2 virus on the delivery of health care for people with spine-related symptoms. Although the SARS-CoV-2 virus was first identified in late 2019, it was not until January and February 2020 that the seriousness of the situation became evident to health authorities globally [1,2]. By March 2020, the world began to recognize the severity of COVID-19 and a lockdown of health care services was considered for all but emergency care. Internationally, public health professionals and government have either mandated or recommended staying at home and physical or social distancing as key interventions necessary to reduce viral transmission. These restrictions have had a dramatic impact on clinicians’ ability to deliver in-person spine care, which traditionally consists of close-contact clinical examination and physical interventions [3,4].

In March 2020, the World Spine Care (WSC) charities [5,6] were forced to close their clinics in all countries where they were operational. This was, in each case, an edict by the health care authorities in the respective countries. Volunteer clinicians had to return home to avoid being stranded in the countries where they were providing services.

During this period, however, people with spine-related symptoms, especially those disabled by these disorders, did not suddenly cease to require care that was simply not immediately available. A series of articles offering advice to clinicians within several specialties that commonly manage people with chronic pain and musculoskeletal and spinal disorders began appearing [7-10]. However, by March and April 2020, no organization had provided guidance that both patients and clinicians with different levels of training and education could follow to triage spine-related symptoms and disability and provide advice on what the current evidence would suggest as a reasonable management strategy for all potential symptom presentations.

In 2018, the Global Spine Care Initiative (GSCI), an initiative of WSC, published 15 articles in a special issue of the European Spine Journal, summarized in an executive summary [11]. The goal of the GSCI was to bring together leading health care providers, scientists, specialists, government agencies, and other stakeholders to review all current guidelines and to develop a care pathway and model of care that was consistent with current evidence and could be used by all clinicians, irrespective of their training and experience. The final articles were the result of collaboration between 68 participants from 24 countries on 5 continents [12]. The publication of the GSCI provided a unique evidence-based foundation to support the development of guides that could be used to support the delivery of spine care during the COVID-19 pandemic.

Physical distancing between clinicians and patients with spine-related symptoms is not limited to restrictions imposed by pandemic-related lockdowns. For people experiencing these symptoms in most low- and middle-income countries, as well as many underserved communities in high-income countries, extraordinarily little if any evidence-based spine care is available and practiced [13].

The objective of this paper is to describe the process of developing and presenting evidence-based patient and clinician guides that can be used for triage, advice, education via telecommunication, and referral for appropriate care when necessary when direct in-person care is not available.

## Methods

The proposed patient and clinician guides were based on 4 principles:

1. Useful during situations where spine care is delivered remotely.
2. Consistent with the recommendations of the most widely accepted evidence-based approach to spinal disorders.
3. Useful for all clinicians who manage spinal disorders, irrespective of their training. This includes family physicians, chiropractors, physical therapists, and medical specialists in such fields as neurology, rheumatology, physiatry, and others.
4. Clear and easily understandable so that patients with different levels of education can reasonably use it, if necessary, with the help of a clinician via telecommunication.

In the end, two sets of guides, one for patients and one for clinicians, were developed by extracting information from the published GSCI papers [14-19]. These papers had reviewed all current, widely accepted guidelines for the management of spinal disorders. The steps recommended in these guides were extracted primarily from the articles on the GSCI Classification of Spinal Disorders [16] and the GSCI Care Pathway [14]. Special attention was paid to the flash cards in the GSCI Care Pathway article. These flash cards provided evidence-based intervention recommendations for each class and subclass of spinal disorder in the GSCI system.

The stimulus for the development of these guides came after a presentation to the Skoll World Forum on April 1, 2020, by World Spine Care, titled “Pain and disability: Making the vulnerable more vulnerable.” This presentation has been viewed by approximately 3000 people. Questions and discussion after this event made it clear that there was a need for information and guidance for the management of spinal disorders during the pandemic. The process of initial extraction and review was completed by two of the lead investigators of the GSCI, a layperson without health care qualifications, and an emeritus dean of an educational institution that graduates clinicians who treat patients with spine-related symptoms.

To ensure a wide consensus and an evidence-based approach that was not limited to members of the GSCI, it was decided that an international and interdisciplinary team would review, provide input on, edit, and modify the guides as necessary. Therefore, 14 individuals who had been coauthors of the GSCI articles and 15 individuals who did not participate in the GSCI were invited to review drafts of the guides. The group included 8 participants who had extensive experience in developing and reviewing evidence-based guides on the management of low back or neck pain; 10 participants who currently held, or had held, senior teaching or administration positions for clinicians who manage spinal disorders in the fields of family practice, chiropractic, and physical therapy; and 15 participants who had extensive clinical experience in family medicine, neurology, physiatry, rheumatology, psychology, chiropractic, physical therapy, and yoga. The participants were from 10 countries on 4 continents.

An open, progressive, iterative approach was used to ensure that every participant would be able to have input and review opinions and recommendations from all other participants. When a participant submitted edits or recommendations, they were incorporated into the guides, with all other participants invited to review and comment on the revisions. A total of 10 versions of the guides were prepared using this process. When there were no further comments or edits from any of the participants, the final draft was converted into a formal educational document for distribution in a professionally produced education format. The process was completed within a 4-week time frame and the final document was posted on the World Spine Care website on April 26, 2020. It has subsequently been posted on the websites of the Society of Indian Physiotherapists and the World Federation of Chiropractic and downloaded over 500 times.

## Results

### The Patient Guide

It was agreed that the patient guide should be limited to a simple 2-step process. The first step requires the patient to describe the nature of their symptoms or concerns. The second step provides the following:

- Information that a patient could use to determine the level of care required for their symptoms.
- Reassurance and recommendations where the evidence suggests that self-care is likely to be of benefit.
- Advice about when it would be important to seek the care of a spine care clinician or to seek emergency care.

It was stressed that not all spine-related symptoms require immediate (clinical) treatment, imaging, or expensive interventions. The following statement was included in the Patient Guide: “You are empowered to self-assess. Your spine care provider is your partner in health, do not hesitate to reach out to them by phone or email if you have questions.”

The first step in the patient guide includes a series of five questions for patients to self-complete that provide adequate information on pain location, severity, and activity impact, and major red flags for serious pathology. Based on feedback from laypeople involved in creating the guides, it was felt that the questions were clear enough that an average patient, even with limited education, could provide answers. Textbox 1 includes the questions that are felt to be essential for this purpose. It was also realized that patients, especially in situations where reading capacity or comprehension might be limited, may require assistance from a clinician, family member, or community helper to answer specific questions. This clarification is included within Textbox 1. The process of using the guides starts with the following statement: “I have back or neck pain or symptoms such as pain, numbness or weakness in my arms or legs or headaches that might be coming from my back or neck. How do I determine the severity of these symptoms?”

**Step 1 of Patient Guide.**

Answer the following five questions to determine whether it is necessary to seek professional help. This can be done personally, or with the help of a licensed clinician or first responder via a telehealth consultation by phone or videoconferencing.

**1. What are my problems/symptoms?**

A. No or minimal discomfort

B. Mild pain

C. Moderate pain

D. Severe pain

E. Numbness or tingling

F. Muscle weakness

G. Loss of balance

H. Recent onset of bladder or bowel problems like loss of control

**2. Do I feel pain beyond my spine?**

A. No

B. Down both legs

C. Down one leg

D. Down both arms

E. Down one arm

F. New or different headaches

G. Chest pain

**3. Are my symptoms stopping me from doing my normal activities?**

A. No. I can do everything

B. Yes, a little. I can do most activities

C. Yes, a lot. I have difficulty doing anything

**4. Have I had a recent fall or accident?**

A. No

B. Yes

**5. Do I have, or have I had, any other serious diseases?**

A. No

B. Cancer

C. Infection such as tuberculosis or HIV/AIDS

D. Osteoporosis or steroid use

E. Any condition that causes inflammation of my joints or muscles

F. Any condition that affects my nerves or brain

The second step in the patient guide is a listing of concerns for which a patient might seek answers. This step includes information that would apply to specific responses from the questionnaire in step 1. The level and severity of the complaints is divided into subgroups consistent with the GSCI Classification of Spinal Disorders [19]: minimal or no discomfort, mild spine pain, moderate spine pain, severe spine pain, symptoms consistent with nerve involvement, possible spine or bone fracture, and possible complications of serious systemic diseases that might cause spine-related symptoms (Textbox 2).

The first question likely to be asked by a patient is whether they need to see a clinician in the office. The answers, as noted in Textbox 2, vary from “not necessary” through “not usually necessary,” “may need,” “may be necessary,” “emergency care is needed,” and “seek care from a medical specialist.” For patients with spine pain and no red flags, self-care recommendations are made that are consistent with current evidence-based guidelines, with emphasis on reassurance and education. This differentiation of symptoms into patterns also ensures that those patients with red-flag symptoms are referred appropriately. It is necessary to ensure that patients who have ongoing concerns can get further information and advice via telehealth communications with a knowledgeable clinician, without having a face-to-face office or hospital appointment.

**Step 2 of the Patient Guide.**

Now that I have described my symptoms, what should I do?

**Minimal or no discomfort and no other symptoms, “Yes” on 1A and “No” on all other questions.**

It is not necessary for you to see a licensed clinician in the office.

If concerned about activities that can cause spine pain, seek information from a reliable source and stay active. Consider a telehealth communication with your licensed clinician if you have questions.

**Mild spine pain, “Yes” on 1B and “No” to all other questions.**

Office-based treatment by a licensed clinician is not usually necessary. Consider self-care recommendations and telehealth communication with a licensed clinician.

Self-care suggestions:

- Keep moving and try to maintain as much of your normal activity as you can.
- Try applying heat or cold over the area of discomfort for 20 minutes maximum. Take care to avoid skin burns if too hot or cold.
- Avoid prolonged sitting or stationary positions.
- Consider home exercises and relaxation techniques such as yoga and tai chi.
- Consider over-the-counter medication such as paracetamol, ibuprofen, and naproxen. These medications should not be taken without first contacting a medical physician if they have caused any prior adverse symptoms or if you have symptoms consistent with the flu.
- Mild spine pain is very common and usually does not become disabling. Usually the pain will improve or resolve within a few days or weeks.
- If the symptoms persist for prolonged period (greater than 6 weeks), consider contacting a licensed clinician who is knowledgeable about spinal disorders.
- Tests such as X-ray and magnetic resonance imaging are not very helpful in the decision of which treatment to consider.

**Moderate spine pain, “Yes” on 1C and 3B.**

Office-based treatment by a licensed clinician is not always necessary. Initially try self-care.

Self-care suggestions:

- Keep moving and try to maintain as much of your normal activity as you can.
- Try applying heat or cold over the area of discomfort for 20 minutes maximum. Take care to avoid skin burns if too hot or cold.
- Avoid prolonged sitting or stationary positions.
- Consider home exercises and relaxation techniques such as yoga and tai chi.
- Consider over-the-counter medication such as paracetamol, ibuprofen, and naproxen. These medications should not be taken without first contacting a medical physician if they have caused any prior adverse symptoms or if you have symptoms consistent with the flu.
- Moderate spine pain is not uncommon. Usually the pain will improve or resolve over time.
- If the pain does not resolve over a period of 2 weeks, or the pain is intolerable, it may be necessary to seek the advice of a licensed clinician for recommendations on how to reduce symptoms.
- X-ray or magnetic resonance imaging or other testing is generally not required unless the pain does not improve over a period of 6 weeks.

**Severe spine pain, “Yes” on 1D and 3C and “No” on all other questions.**

Office-based treatment by a licensed clinician is not always necessary. Initially try self-care.

Self-care suggestions:

- Keep moving and try to maintain as much of your normal activity as you can.
- Try applying heat or cold over the area of discomfort for 20 minutes maximum. Take care to avoid skin burns if too hot or cold.
- Avoid prolonged sitting.
- Consider over-the-counter medication such as paracetamol, ibuprofen, and naproxen. These medications should not be taken without first contacting a medical physician if they have caused any prior adverse symptoms or if you have symptoms consistent with the flu.
- Severe spine pain is less common. Even severe spine pain, in most cases, in the absence of major injury, nerve symptoms, or serious disease, tends to improve or resolve with time.
- If the pain is intolerable, it may be necessary to seek the advice of a licensed clinician for recommendations on whether you should be seen in an outpatient setting, require testing, and how to reduce symptoms.
- X-ray or magnetic resonance imaging or other testing may be necessary.

**Symptoms consistent with nerve involvement: pain, numbness, or tingling in arms or legs (“Yes” to 1D and/or 1E). Muscle weakness (“Yes” to 1F). Loss of balance (“Yes” to 1G). New onset of bowel or bladder problems (“Yes” to 1H). Severe new onset of headaches or chest pain (“Yes” to questions 2B, 2C, 2D, and/or 2E).**

Office-based treatment by a licensed clinician may be necessary.

- If symptoms are of recent onset (less than one week), consider contacting a health care practitioner or emergency room for a detailed examination.
- If you have experienced recent onset of incontinence, loss of bowel or bladder function, or marked loss of balance, muscle weakness, or difficulty walking, go to the emergency department. The licensed clinician or emergency physician will determine if you require X-ray or magnetic resonance imaging, or other testing and treatment.

**Possible spine/bone fracture, severe fall, or accident with severe spine pain (“Yes” on 1C and 4B).**

Emergency treatment is necessary.

- Have someone call for an ambulance or nearest help.
- Keep still and do not move.

**Possible complication of a serious problem that is affecting the spine, “Yes” on any of the conditions noted in question 5.**

Seek care from a licensed clinician or medical specialist to determine whether your serious disease is causing your spine-related symptoms. Diseases such as cancer, certain infections, and inflammatory rheumatologic diseases can impact the spine and cause pain.

### The Clinician Guide

The consensus of the panel was that a clinician guide should be clear and useful to all clinicians, irrespective of their professional qualifications and experience, as well as those who may not be familiar with spine care guidelines. It should be consistent with accepted evidence-based clinical practice. It should be sufficiently comprehensive to allow for documentation of the clinician thought process and ensure adequate medical record keeping, while at the same time not be overly time consuming. A 5-step process was recommended:

1. Obtain and document patient demographics, location of primary clinical symptoms, and psychosocial information. The guide recommends a minimum of the following: patient name, date, age, sex or gender, normal occupation, current work situation, worker’s compensation status (if applicable), location of primary complaint, comorbidities, level of anxiety and concern about the condition, and psychiatric history. A downloadable page with these questions is made available but it is recognized that clinicians may wish to develop their own charting method and include additional information on patient demographics or take a more comprehensive psychosocial history.

2. Ensure that the questionnaire recommended in the Patient Guide (Textbox 1) has been completed. The clinician should complete this questionnaire with the patient if it is incomplete and review the answers with the patient to ensure that the questions are understood and answered correctly. Clinicians may also want to quantify the patient’s level of pain and disability to a greater extent using visual analog scale (VAS) scores and the NIH Pain Consortium Impact Classification (NIH PCIC) score [20], as recommended in the GSCI publication [19]. The values for each of the questions on pain and disability are consistent with the terms minimal, mild, moderate, and severe symptoms as noted in the Patient Guide. Rating scales are not included in the Patient Guide. It is also noted that any valid disability scale that provided similar differentiation of levels of disability could be used instead of the NIH PCIC score.

3. Determine the classification of the patient’s complaints according to the GSCI Classification of Spinal Disorders (Textbox 3, Major Classes). The nomenclature of the GSCI classification system is consistent with the “minimal” through “severe” terminology noted in the patient guide. In the Patient Guide, the patient’s definition and judgment of the terms “minimal” through “severe” is used. The class of spinal disorder in the Clinician Guide requires consideration of the VAS score, NIH PCIC score, or a similar disability score, together with questioning of the patient to understand what these terms mean to them.

**Steps 3 and 4 of Clinician Guide.**

Step 3 of Clinician Guide: What GSCI class of back or neck pain best represents the patient’s symptoms?

Step 4 of Clinician Guide: Determination of GSCI spinal disorder subclassification that best describes the patient presentation [16].

**Class 0: minimal or no discomfort but no other symptoms. “Yes” on 1A and “No” on all other questions.**

- Subclass 0a: no history of risk factors
- Subclass 0b: history of risk factors

It is not necessary to see a clinician in his or her office. Telehealth may be considered.

**Class I: mild spine pain. “Yes” on 1B and “No” to all other questions.**

- Subclass Ia: acute (duration <3 months)
- Subclass Ib: chronic (duration >3 months)

Office-based treatment by a clinician is not usually necessary. Telehealth may be necessary. Advice and reassurance may be helpful.

**Class II: moderate or severe spine pain. “Yes” on 1D and 3C or 3D and “No” on all other questions.**

- Subclass IIa: acute (duration <3 months) moderate pain and disability
- Subclass IIb: chronic (duration >3 months) moderate pain and disability
- Subclass IIc: acute, severe pain
- Subclass IId: chronic, severe pain and disability

IIa and IIb: Office-based treatment by a clinician is not always necessary. Telehealth is important. Advise and reassure. Regular telehealth follow-up may be necessary.

IIc: Office-based treatment by a clinician may be necessary. In many cases, acute symptoms can be managed via telehealth in the absence of red flags.

IId: Office-based treatment by a clinician may not be necessary unless there is a flare-up of incapacitating symptoms. Consider telehealth consultation first.

**Class III: symptoms consistent with nerve problem. Pain, numbness, or tingling in arms or legs, new onset marked muscle weakness, and/or new onset of bowel or bladder problems (“Yes” to 1D and/or 1E and/or 1F and/or 1G and/or 1H). Severe new onset of headaches or chest pain (“Yes” to 2B and/or 2C and/or 2D and/or 2E). Consider additional questions on gait difficulty, loss of balance, and/or loss of hand function (including clumsiness and change in dexterity) that may represent symptoms of myelopathy.**

- Subclass IIIa: minor or nonprogressive
- Subclass IIIb: acute, major or progressive
- Subclass IIIc: chronic and stable

Office or emergency room treatment by appropriate clinician is necessary if the symptoms are acute or progressive.

**Class IV: possible spine/bone fracture. Severe fall or accident with severe spine pain (“Yes” on 1C and 4B).**

- Subclass IVa: stable spine structural pathology with no serious symptoms or red flags. Office-based treatment is not necessary. Telehealth may be considered for advice and reassurance.
- Subclass IVb: acute (eg, fracture) or chronic (eg, instability) spine structural pathology that correlates with symptoms. Emergency treatment is necessary.

**Subclass V: possible complication of a serious problem that is affecting the spine. “Yes” on any of the conditions noted in question 5.**

- Subclass Va: severe acute spine pathology. Requires immediate attention (emergency).
- Subclass Vb: slowly progressive spinal pathology. Requires intervention (nonemergency).
- Subclass Vc: symptoms originating from nonspinal pathology. Requires immediate attention (emergency). Referral to patient’s medical family/general physician or specialist to determine whether serious disease is causing the patient’s spine-related symptoms. Advise patient if emergency attention is required.

4. Determine the GSCI subclassification of the symptom pattern. This requires three additional questions.

Question 1 is the determination of the duration of the symptoms. Most spine guidelines recommend different interventions for acute versus chronic spine-related symptoms [21,22]. The differentiation between these terms varies somewhat in review articles and guidelines, with acute being defined as a few days to a few weeks and sometimes subdivided into acute (1-4 weeks) and subacute (4-12 weeks) [21,23]. It was elected to use the recommendations of the NIH Task Force on Research Standards, which defines chronic low back pain as present for >3 months and present for at least half the days in the past 6 months [20]. In specific patients, especially in the red flag classifications, acute may involve a very recent onset of symptoms and may require immediate or emergency care (1 day to 1 week).

Question 2 is the determination as to whether symptoms are progressive or stable. Again, these subclasses are most important in the red-flag and neurologic symptom classifications but also are useful in determining how to manage pain symptoms.

Question 3 includes enquiries on risk factors for spine pain and comorbidities. The information gained from this question allows the clinician to determine the presence of psychosocial factors known to impact disability, but also can be important for the understanding of a patient’s general health and activity level and whether comorbidities are controlled. The subclassifications are noted in Textbox 3 under each of the major classes.

5. Treatment interventions. It was not possible to list the extensive number of treatment recommendations for each class of spinal disorder. The clinician is advised to review the GSCI flash cards for each class and subclass of spinal disorder [19]. Clinicians are also advised to refer to websites presenting leading government guidelines [21,24] and those developed by major professional spine care societies, including guidelines specifically adopted by family physicians [22,23], chiropractors [25], and physical therapists [26] and which are felt to be reasonably consistent with each other. Lead statements on the importance of self-care, advice and reassurance, and exercise, as well as potential adverse events from pharmaceuticals, are included. A further statement stresses that imaging is rarely helpful in spinal pain complaints in the absence of other symptoms or red flags. Finally, it was felt that patients should be empowered to embrace self-care but should be made to feel that they are being cared for through follow-up contact with their clinician when they have questions or concerns (Textbox 4).

The Clinician Guide includes a textbox that provides guidance on some of the legal and ethical issues related to telehealth that may not be immediately clear to a practicing clinician (Textbox 5). These include licensing, malpractice, patient informed consent and patient record keeping and confidentiality (Textbox 5).

**Step 5 of the Clinician Guide: treatment considerations.**

On completion of the questionnaire and determination of the class and subclass of spinal disorder, consider the following:

1. Follow World Health Organization, US Central Disease Control, UK National Health Service, and other national government agency guidelines for the current status and recommendations regarding the prevention and management of COVID-19.

2. Consult national evidence-based treatment guidelines for evidence-based treatment options for each subclass of spinal disorder. Consider the Global Spine Care Initiative flash cards for a review of these guidelines and the interventions recommended for each class and subclass of spinal disorder.

Other resources:

- UK National Health Service Back Pain Guidelines
- American College of Physicians Clinical Practice Guideline
- Canadian Chiropractic Guideline Initiatives on Neck Pain, Low Back Pain, Self-Management Resources, Exercise Videos
- American Physical Therapy Association Clinical Practice Guidelines
- Task Force on Neck Pain Executive Summary

3. Advise patients over the phone, provide video consultations (in some regions partially or fully reimbursed by health insurance), and use social media including Facebook to educate patients. Considerations include the following:

- Research existing online educational media that satisfies the requirements for each individual patient.
- Consider leading online educational classes with patients.
- Consider leading or referring to online yoga, including the World Spine Care Yoga Project, tai chi, Pilates, or rehabilitative exercise group classes with patients.

4. For Class 0, Class I, and moderate Class II, reinforce that the current research suggests that self-care is usually enough to control symptoms. Provide advice and reassurance that may relieve the pain and aid recovery.

5. The taking of over-the-counter medication including paracetamol and nonsteroidal anti-inflammatory drugs (NSAIDs), including ibuprofen and naproxen, is recommended for short-term relief of back and neck pain by most evidence-based guidelines. Patients should be aware of potential adverse events, including gastrointestinal bleeding and ulcers, and cardiovascular and renal disorders. The possibility that NSAIDs may negatively impact COVID-19 pulmonary symptoms has been raised but not confirmed at the time of publication [27]. Clinicians should be able to advise patients or refer patients to a reliable source of information when asked by patients about taking these medications.

6. Reinforce that testing such as X-ray and magnetic resonance imaging rarely help inform the decision of which treatment to consider. Referral to a surgeon is unlikely to be necessary unless the patient is incapacitated, has had a significant injury, or has red flags that result in Class III, IV, or V assessment.

7. Be available for regular follow-up contact with the patient (virtually).

8. Recognize that patient symptoms on rare occasions can get worse, so that they could eventually fall into a different class or subclass and require different or more immediate care than originally recommended.

9. Patients should be empowered to embrace self-management but still feel they are being cared for.

**Clinician Guide for telehealth considerations.**

1. Find out if telehealth communication is allowed in your jurisdiction.

2. In many countries and regional jurisdictions, including the United States, be careful about providing telehealth sessions to patients who live outside of your jurisdiction. You may be sanctioned for practicing without a license. Follow all legal recommendations for telehealth.

3. Check with your malpractice carrier to make sure there are no restrictions for coverage while conducting telehealth sessions.

4. In addition, make sure to obtain a verbal consent from the patient in which they acknowledge there are limitations to a telehealth session, which is not the same as a physical examination (eg, it is not possible to do a detailed neurological examination on the phone).

5. Be careful not to violate any patient privacy rules. Use a video program or platform that provides an appropriate level of patient confidentiality to satisfy regional or national laws.

6. Find out if compensation is allowed, including billing requirements that may necessitate mandatory documentation regarding telehealth sessions with your patients.

7. Keep records of your consultation.

8. Make sure to get the address of the physical location of the patient. This may be necessary in case an emergency arises during the telehealth session.

The Clinician Guide concludes with a section that has the following heading: “During this period of social and physical distancing what precautions must I consider when seeing a patient while protecting both the patient, me, and my staff from infection?” This section provides guidance that is widely recommended by the World Health Organization (WHO), Centers for Disease Control and Prevention (CDC), the UK National Health Service (NHS), and other government agencies based on the geographical area of practice. It is noted that these guidelines are fluid and clinicians should stay informed of best practices and requirements in their jurisdictions.

The Patient Guide and Clinician Guide can be downloaded in their professionally produced educational formats [28,29].

## Discussion

### Principal Results

The guides recommended in this article have been developed by an international team from 10 countries on 4 continents. Care was taken to have a large panel of 29 participants that included members from most of the nonsurgical spine care professions and specialties as well as patients. The process of developing these guides was an open iterative approach that allowed all participants to edit, comment on, or modify the recommendations. The guides are therefore not dependent on the recommendations from a single published spine care guideline, the opinions of a small number of clinicians or scientists, a single culture or part of the world, or a single spine care profession or discipline. These guides were extrapolated from a recent review of the most current international spine care guidelines, which were published in September 2018 [11]. The Patient and Clinician Guides minimize any potential conflicting information given to patients and their clinicians. Finally, these guides cover the entire spectrum of spinal disorders from minimal pain or concerns, through mild, moderate, and severe spine pain, neurological symptoms, trauma, and systemic diseases that can result in spine-related symptoms.

### Comparison With Prior Work

The Patient and Clinician Guides described in this article are not the first or only guides that have been developed to help clinicians and patients during the current COVID-19 pandemic. The current COVID-19 pandemic has generated a great deal of interest and a number of articles that have focused on the difficulties of managing patients with chronic pain when in-person care is not feasible [8,9,30]. Most of these articles have focused on the challenges and terminology, or different telehealth or eHealth tools. There are also a number of articles that address specific subgroups of patients with spinal disorders such as low back pain [31], general musculoskeletal disorders [7,10,32,33], and inflammatory joint diseases [34], as well as triage of surgical patients [3]. Many of the articles addressing spine care during COVID-19 make recommendations based on a single or unspecified guideline [35]. We are not aware of any specific international, interdisciplinary comprehensive spine care guide that has been developed for use during a pandemic where lockdown restrictions have limited in-person care. None that we have found have addressed the full spectrum of spinal disorders with recommendations for both patients and clinicians.

### Future Considerations

The COVID-19 pandemic and resulting limitation of in-person contact between patients and their clinicians will come to an end at some point. We are confident that the utility of these guides will persist after the pandemic and that the new normal will make greater use of virtual spine care interactions. This is likely to be more important where there are geographically dispersed populations or where spine specialists are not available, accessible, or affordable.

People with spine-related complaints or disabilities are rarely receiving care that is consistent with current evidence-based guidelines. This is true in high-, middle-, and low-income countries [13].

In high-income countries, the problem in the delivery of spine care is widely considered to be overutilization of many costly yet low-value interventions such as indiscriminate use of imaging, opioids, and invasive procedures [36]. The broad usage of the proposed linked guides for clinicians and patients ensures similar assessment and management options. This has the potential to reduce the overutilization of unnecessary and expensive interventions while increasing the empowerment of patients to self-manage uncomplicated spinal pain with the assistance of their clinician, either through direct in-person consultation or via telehealth communication.

In low- and middle-income countries, physical distancing between patients and clinicians trained and capable of providing evidence-based care is not available for most of the public. Foster et al [13] pointed out in their review of the literature that in many countries, the primary management strategy for patients who present with spine pain is either emergency room care, admission to hospital, or direct referral to an orthopedic surgeon. Irving et al [37] noted that for 50% of the world population, primary care physicians spend less than 5 minutes of face-to-face time with patients during a consultation. The most common intervention for back pain is limited to the prescription of NSAIDs or opioids. The likelihood that primary care or family practice clinicians could find the time to offer evidence-based spine care—which requires a clinical assessment and includes education, reassurance, and advice on lifestyle and exercise, as well as symptom relief—is remote. For much of the world, there is a dearth of any effective care by nonsurgical clinicians who have specific training and an interest in managing patients with spinal disorders. The proposed guides have the potential to reduce this deficit.

### Telehealth Considerations

This lack of alternatives raises the possibility that patient and clinician guides such as those presented in this paper and those developed by local agencies (eg, the Ontario Inter-professional Spine Assessment and Education Clinics [38]) could be used to connect patients with clinicians knowledgeable about the management of spinal disorders through telehealth. Such tools have the potential to improve care and reduce the load on emergency rooms, hospitals, and advanced specialists and surgeons without burdening family and primary care physicians. Patients would have access to information and the ability to consult virtually with a knowledgeable clinician. This will hopefully result in improved outcomes and well-being. There is early evidence that telehealth [39] or digital care can have a positive impact on low back pain [39-41] and that these modalities are well accepted by patients, at least in conjunction with in-person access to a clinician when needed. The more general use of an app to educate patients with musculoskeletal pain is also being explored [32]. This is becoming more feasible with the extensive and increasing availability of access to mobile phones in many low- and middle-income countries [42,43].

Telehealth is an expanding field with increasing understanding of what is possible through this medium for both acute and chronic consultations [43]. There are now several publications that describe a virtual neurologic examination [44,45]. There is also a growing number of publications addressing the possibility of virtual spine examination [46,47,48], although none of these virtual examinations have been studied for validity or reliability.

### Limitations

The GSCI classification was developed through an iterative, broad consensus process and has not been validated through clinical trials at the time of writing. Although the guides were developed with extensive international and interprofessional representation, there is underrepresentation from Asian, Middle Eastern, and Western European countries, which may reduce their acceptance or compatibility with cultural norms in these and other regions of the world. Future revisions of these guides would benefit from input from wider geographic and cultural representation. Work is currently underway to test the GSCI model of care in three underserved communities around the world. Studies are also being proposed to test these guides to determine their acceptance by patients and clinicians, the mechanism through which these guides can be implemented, and their impact on patients with spinal disorders. It is also necessary to determine whether these guides influence the use of unnecessary, costly, and often ineffective interventions that do not comply with current evidence-based guidelines. The consideration of guides for spine-related disorders is a new and expanding field and the recommendations noted in Textboxes 2 and 3 should be considered fluid and are likely to change as our experience with telehealth guides increases.

### Conclusion

This article describes the development of patient and clinician guides for the management of patients with all levels of spine-related symptoms who are, for various reasons, unable to access/conduct in-person office-based care. The guides were developed by an international, interdisciplinary panel of 29 participants from 10 countries, with the goal of providing guidance to both patients and their clinicians in situations involving physical distancing. The initial goal was to fill the gap in care during the COVID-19 pandemic lockdown period. It is believed, however, that these guides may be helpful in other situations where there is limited availability of evidence-based care in underserved communities. They should provide patients with both guidance and alternative options for access to spine care clinicians either digitally or via telehealth.

## Abbreviations

CDC: Centers for Disease Control and Prevention
GSCI: Global Spine Care Initiative
MRI: magnetic resonance imaging
NHS: National Health Service
NIH: National Institutes of Health
NIH PCIC: National Institutes of Health Pain Consortium Impact Classification
NSAID: nonsteroidal anti-inflammatory drug
PCIC: Pain Consortium Impact Classification
VAS: visual analog scale
WHO: World Health Organization
WSC: World Spine Care
